# Skeletal Fluorosis Mimicking Spondyloarthritis: A Rare Presentation

**DOI:** 10.31138/mjr.33.2.261

**Published:** 2022-06-30

**Authors:** Prasanta Padhan, Debashis Maikap

**Affiliations:** Department of Clinical Immunology and Rheumatology, Kalinga Institute of Medical Sciences, KIIT University, Bhubaneswar, Odisha, India, PIN: 751024

**Keywords:** fluorosis, spondyloarthritis, osteosclerosis, calcification

## CLINICAL IMAGE

**Figure 1.**
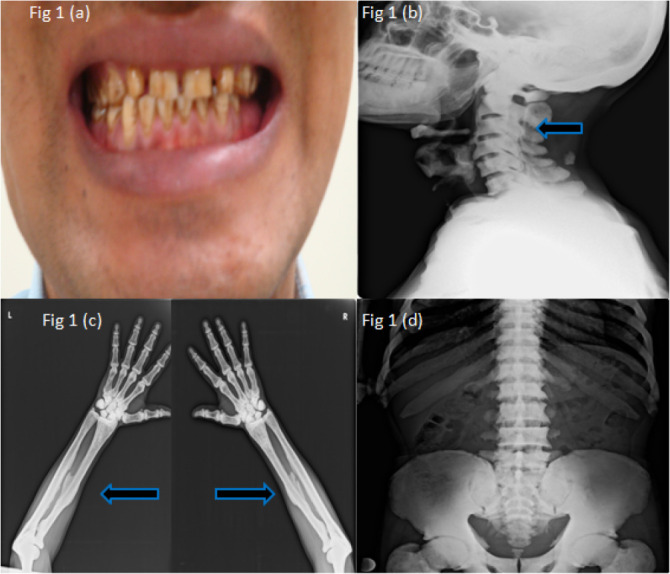
**(a)** Teeth showing dark brown streak and discoloration with pitted and rough enamel. **(b)** X-ray of skull and cervical spine showing osteosclerosis and the posterior longitudinal ligament ossification with calcification of thyroid and cricoid cartilage. **(c)** X-ray of both forearms showing interosseous membrane calcification. **(d)** X-ray of the pelvis showing osteosclerosis of the vertebral column, exuberant calcification of pelvis and greater trochanter along with prominent hypertrophic spurring at the acetabular margins.

A 46-year-old male presented to us with a 7-year history of pain in neck and lower back with recent onset pain in knees, elbows, and heels for the past one year. He was being treated for spondyloarthritis with intermittent analgesics for the past one year without benefit. On examination, there was brownish discoloration of teeth with rough and pitted enamel (**Figure 1A**). Musculoskeletal system examination showed tenderness on right elbow and bilateral achilles tendinitis. Spine examination revealed diffuse tenderness at the cervical spine, and the lumbo-sacral spine with mild restriction of forward and lateral movement. His modified Schober’s test was 3cm and FABER test was negative. Laboratory investigation showed raised ESR (36 mm/1^st^ hr; Normal <20 mm/1^st^ hr and CRP (12mg/L; normal <5 mg/L). His complete blood counts, serum electrolytes including calcium, phosphorous, magnesium, alkaline phosphatase, Vitamin D3, and parathormone levels were normal. His rheumatoid factor, anti-CCP (anti-cyclic citrullinated peptide), and human leukocyte antigen B27 (HLA B27), test was negative. Plain radiograph of the pelvis showed osteosclerosis of the vertebral column, exuberant calcification of pelvis and greater trochanter along with prominent hypertrophic spurring at the acetabular margins (**Figure 1D**). A plain radiograph of forearm bones was done, which demonstrated calcification of bilateral interosseous membranes of forearm bones. Skull and spine radiograph showed osteosclerosis and posterior longitudinal ligament ossification with calcification of thyroid and cricoid cartilage of the neck (**Figure 1B,C**). His serum fluoride (F) level was 0.2 ppm (normal range <0.02 ppm) and his 24-hour urine fluoride level was 0.9 (normal <0.10 ppm) confirming diagnosis of dental and skeletal fluorosis. Our patient hailed from endemic area of fluorosis in Odisha, a state of India.

Other aetiologies for non-endemic fluorosis include chronic excessive consumption of black tea, recreational inhalation of fluoride containing vapours (huffing fluoro-carbons), exposure to computer duster containing fluoride such as di- or tetrafluoroethane.^1,2^ Skeletal fluorosis can mimic various arthritis such as rheumatoid arthritis, osteoarthritis or spondyloarthropathy.^3^ The early stages of the disease may be asymptomatic or present as vague pain in the neck or back with rigidity, arthralgia and paraesthesia in the limbs,^4^ achilles tendinitis, and early morning stiffness.^5^ The present case had skeletal fluorosis resembling spondyloarthritis. Awareness of clinical, radiographic, and dental findings of fluorosis can help the physicians and rheumatologists from endemic areas to make early diagnosis and unnecessary workup.
